# Modeling the Emergence of Contact Languages

**DOI:** 10.1371/journal.pone.0120771

**Published:** 2015-04-15

**Authors:** Francesca Tria, Vito D.P. Servedio, Salikoko S. Mufwene, Vittorio Loreto

**Affiliations:** 1 Institute for Scientific Interchange (ISI), Via Alassio 11C, 10126 Torino, Italy; 2 Institute for Complex Systems (ISC-CNR), Via dei Taurini 19, 00185 Roma, Italy; 3 Sapienza University of Rome, Physics Dept., Piazzale Aldo Moro 5, 00185 Roma, Italy; 4 University of Chicago, Dept. of Linguistics, 1115 E. 58th Street, Chicago, IL 60637, USA; 5 SONY-CSL, 5, Rue Amyot, 75005, Paris, France; University of Maribor, SLOVENIA

## Abstract

Contact languages are born out of the non-trivial interaction of two (or more) parent languages. Nowadays, the enhanced possibility of mobility and communication allows for a strong mixing of languages and cultures, thus raising the issue of whether there are any pure languages or cultures that are unaffected by contact with others. As with bacteria or viruses in biological evolution, the evolution of languages is marked by horizontal transmission; but to date no reliable quantitative tools to investigate these phenomena have been available. An interesting and well documented example of contact language is the emergence of creole languages, which originated in the contacts of European colonists and slaves during the 17th and 18th centuries in exogenous plantation colonies of especially the Atlantic and Indian Ocean. Here, we focus on the emergence of creole languages to demonstrate a dynamical process that mimics the process of creole formation in American and Caribbean plantation ecologies. Inspired by the Naming Game (NG), our modeling scheme incorporates demographic information about the colonial population in the framework of a non-trivial interaction network including three populations: Europeans, Mulattos/Creoles, and Bozal slaves. We show how this sole information makes it possible to discriminate territories that produced modern creoles from those that did not, with a surprising accuracy. The generality of our approach provides valuable insights for further studies on the emergence of languages in contact ecologies as well as to test specific hypotheses about the peopling and the population structures of the relevant territories. We submit that these tools could be relevant to addressing problems related to contact phenomena in many cultural domains: e.g., emergence of dialects, language competition and hybridization, globalization phenomena.

## Introduction

By definition, one speaks of contact languages [1–3] whenever more than one language are in use in the same place, at the same time, and within the same collective population [2]. From this perspective the notion of contact language is very general and it includes all cases of bilingualism, the coexistence of dialects with the official language, all situations in which a *lingua franca* is needed and of course all the examples of creoles and pidgins [4]. It is noteworthy that the number of examples of contact languages is exploding, also thanks to the extraordinary opportunity for mobility and communication that modern Information and Communication Technologies (ICT) allow. One of the interesting aspects of contact languages concerns the time-scales of their emergence and evolution. Those time-scales could be extraordinarily short in comparison with those involving many generations. This makes contact languages an ideal playground to investigate linguistic and cultural phenomena [5]. In this paper we focus on the emergence of creole languages as a poster case.

Creole languages [4, 6–8] offer an invaluable opportunity to study the processes leading to the emergence and evolution of Language, thanks to the short—typically a few generations—and reasonably well defined time-scales involved in their emergence. In many well-documented cases, creoles emerged in large segregated sugarcane or rice plantations on which the slave laborers were the overwhelming majority. Lacking a common substrate language, slaves were naturally brought to shift to the economically and politically dominant European language (often referred to as the lexifier) to bootstrap an effective communication system among themselves. Some of the best known examples include Gullah and Jamaican creole (lexified by, i.e., deriving most of their vocabularies from, English) as well as Haitian and Louisiana creoles (lexified by French).

The language transmission process happened despite the limited direct interactions between the slaves and the colonists after the homestead phase, eventually leading to the emergence of creole languages. Even when the social conditions were different from the ones just depicted, one refers to a creole language variety as a language, or vernacular, that emerged in a specific contact ecology hosting two or more languages, a lexifier and one or more substrates [9, 10]. The relevance of the study of creole languages to that of other languages [11, 12] has perhaps been underestimated so far [10, 13], also in relation to the fact that the genetic classification of creoles goes beyond the tree models often used in genetic linguistics [14–17], calling for methods that are able to account more adequately for varieties produced by language mixing. Also, the particular economic poverty conditions of creole language areas (e.g. Haiti, Jamaica, Guyana, Sierra Leone, Cape Verde, etc.) have historically contributed to considering these vernaculars as degenerate outcomes of European languages and to studying them from the perspective of “creole exceptionalism” [18] (i.e., they evolved in their own unusual or exceptional way compared to other languages considered more normal). However, such ecological specificities, in regard to which even creoles differ among themselves, do not mitigate the fact that the process of language formation applying to creoles is far more general. For instance, as explained in [13, 19], an extensive language shift is also at the origin of the Romance languages, where Celtic populations gave up their languages for Vulgar Latin (although over a remarkably longer time scale and under different socioeconomic contact conditions than those that produced creoles) and modified it into new vernaculars.

Although many studies have been devoted to creole languages (see for instance the recent Atlas of Pidgin and Creole Language Structures [4]), the dynamical processes leading to their emergence remain controversial and poorly understood. In this essay, we model this aspect of their emergence, hoping to shed light on some specific ecological conditions that favored them. We model the process of language restructuring with divergence, which applies also to non-creole language varieties, in contact ecologies involving especially European colonists and African slaves in the 18th and 19th centuries, based on available census data. We address some issues arising from our modeling by articulating specific peopling patterns and population structures, which suggest ways in which modeling can be used as a research tool to clarify accounts of where creoles emerged and what specific ecological factors explain why they did not emerge elsewhere.

We assume that in all the relevant territories Europeans, Mulattos and Bozals cohabited in social conditions characterized by a population structure with several degrees of segregation. (We will use the term “Mulatto” in this essay, more often than the term “Creole”, which has been found more relevant to creole studies, because it is the one that occurs in the census data that we used for our modeling. We do not want to suggest that only Mulattoes of the homestead phase appropriated the European language as their vernacular.) In these ecologies the European language was appropriated as the vernacular of the Mulattos; and the variety they produced was emulated and restructured by the Bozal slaves, in a population structure that favored the influence of substrate languages. These are the languages that the Africans brought with them but could not maintain because they found themselves in ecologies that disfavored their use.

## Materials and Methods

### Insights from census data

The Census data were collected on-line. They report the demographics of Europeans (Whites), Free Colored (Mulattos) and Blacks (Bozals). (We use these categories for convenience sake, although they do not literally correspond to those used in creole studies). Those for several States of USA can be found at *http://www.census.gov/history*, while for Central America and the Caribbean can be found at *http://www.jamaicanfamilysearch.com/Samples/1790al11.htm*.

**Fig 1:**
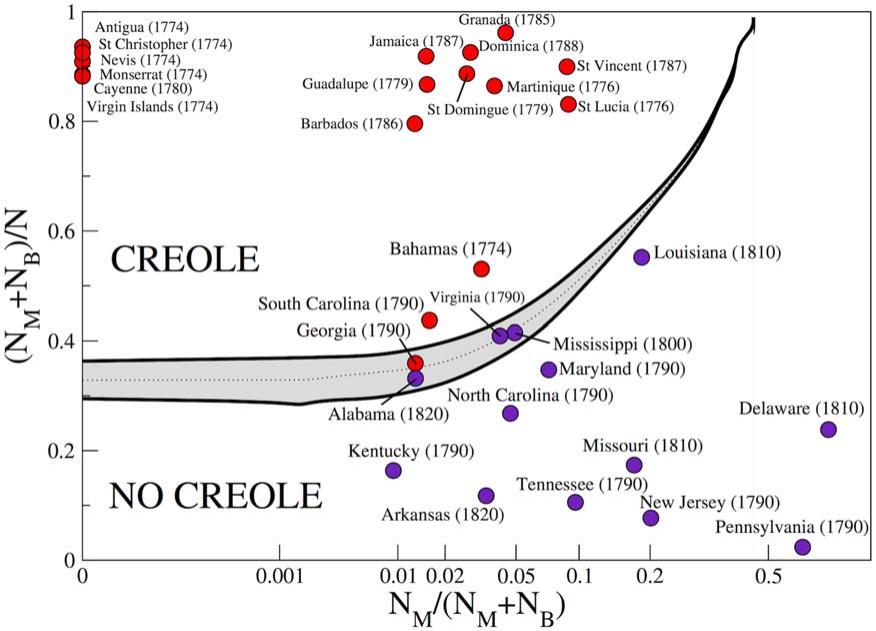
Clustering in the creole formation process. Points are the projection of the census data (See Tables A-D in the S1 Supporting Information) in the plane (*N*_*M*_/(*N*_*B*_+*N*_*M*_), (*N*_*M*_+*N*_*B*_)/(*N*_*M*_+*N*_*B*_+*N*_*Eu*_)). Red circles mark States where a creole language emerged while purple ones identify States where a creole language historically did not emerge. The gray stripe is the outcome of our modeling scheme and separates the regions where respectively the creole *C* (above the stripe) and the European *E* (below the stripe) represent the dominant language (i.e., spread among more than the 80% of the population) in the Mulattos and Bozal populations in the asymptotic states of the model. The two black curves delimiting the gray stripe and the dashed line in the middle are obtained by simulations performed with the same parameters *γ* = 0.8, *δ* = 0.1 and *N* = *N*_*M*_+*N*_*B*_+*N*_*Eu*_ = 10000, with *ε* ranging from 0.05 (bottom black curve) to 0.07 (upper black curve), passing through 0.06 (dashed curve). The horizontal axis has been artificially expanded by a power 0.2.

A first observation pointing to the relevance of the population structure to creole emergence comes from demographic data at the beginning of the creolization process, i.e. between the 18th and 19th centuries AD. Remarkably, on the basis of census data alone, it seems possible to make predictions on the emergence of creole languages. Although the situation is in reality not so straightforward, as we will show in details in the S1 Supporting Information, the demographic composition coupled with relevant historical data turns out to account fairly accurately for the emergence or non-emergence of creole. In particular, we refer to census data about the latest decades of the 18th century and the beginning of the 19th, reporting the number of Free Whites (or European *Eu*), Free Creole Blacks (mainly Mulattos *M*), and Bozal Blacks *B* in several States of USA and the Caribbean (as explained below). We identify two relevant parameters as the fraction of Mulattos in the Black population (*N*_*M*_/(*N*_*B*_+*N*_*M*_)), and the fraction of Blacks in the overall population ((*N*_*M*_+*N*_*B*_)/(*N*_*M*_+*N*_*B*_+*N*_*Eu*_)), such that, by looking at the census data in a bidimensional space whose coordinates are precisely the above mentioned fractions, one can identify a clear separation between places where historic creoles have emerged and places where no creole has formed (Fig 1).

Fig 1 provides a very interesting indication that points to a crucial role social structures could have played during the emergence of creole languages. Can we make this observation more quantitative? In particular, can we ground the empirical observations in the framework of a solid modeling scheme, simulating the creation of a new language variety out of the contact of two or more preexisting ones? These are the questions we now focus on in the attempt to shed some light on the dynamical mechanism leading to creoles formation, giving at the same time a quantitative account for the observations above.

### A simple modeling scheme

**Fig 2:**
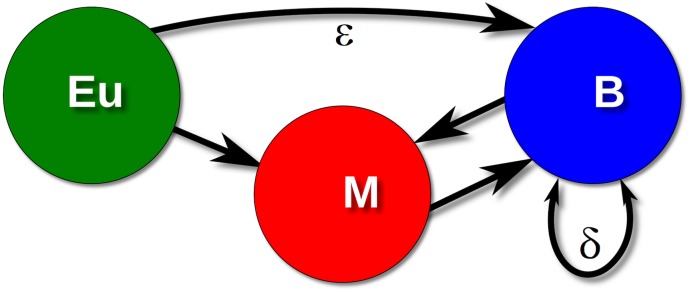
Interaction topology. Europeans (*Eu*), can speak only in their European language to Mulattos (*M*) and with probability *ϵ* to Bozal (*B*). Mulattos and Bozal communicate among them both as *speakers* and *hearers* (refer to the Materials and Methods section). They can speak European (*E*), African (*A*), or the emergent creole (*C*). As noted above, we cannot yet get in the multitude of languages spoken by the Bozal slaves and simply represent the set of languages as a unique language *A*. However, to model in a coarse grained fashion the African multilingualism, we introduce a parameter *δ* that accounts for the possibility that a Bozal slave would success to communicate with another in an African language (refer to the Materials and Methods section for details).

With this aim in mind, we judged the Naming Game (NG) [20] framework as particularly suitable for this task since it simulates how a population of individuals can bootstrap linguistic consensus [21–24]—on cultural timescales—out of the local interactions of pairs of individuals in a population. (For a review of social dynamics see [5].) We stress that our aim here is not that of entering in the details of the creole formation at a fine-grained linguistic level, rather that of uncovering some of the general mechanisms that determine the emergence of contact languages, and that successfully apply to the case of creole formation. In particular, we here refer to a variant [25] of the NG [20] in which bilingual individuals, i.e., individuals familiar with two different languages, may end up developing a third language. It is important to remark that here we make a specific hypothesis that deviates from the original Naming Game model: the model we consider is not a reference game, since there are no objects to be named. Rather the inventory of each individual includes, at a coarse grained level, the list of languages she is able to speak. Depending on success or failure in communication, each individual can slightly shift her inventory of languages eventually adopting a brand new language. If one individual is exposed to two different languages, e.g., her mother tongue and the colonizer’s language, she could start a series of linguistic processes leading to an hybridization of the languages he/she knows. Of course this hybridization will be relevant only because it can affect the whole population, making the hybridization process widespread. The ensemble of all these processes can be represented, at a coarse-grained level, as an effective probability that, in each individual inventory, the pair of languages can be substituted with a brand new language. Within this framework, we superimpose to this simple modeling scheme a specific contact ecology, to address the question of which population structure favors or disfavors the emergence of a creole language. We know that the cradle of creole language formation is a segregated population, in which Whites and non-Whites lived separately and did not socialize together, though they met at the work place. Therefore, oversimplifying the scenario somewhat, we consider three different sets of populations: Free Whites, Free Blacks, i.e., mainly Creole Mulattos, and Bozal slaves, and we adopt an interaction topology by assuming a segregation regime. More specifically, Europeans are treated in a simplified way as an endless and unchangeable source of the European lexifier *E*, never changing their language (the cases where the Europeans who interacted regularly with non- Europeans also learned to speak creole as the only or other vernacular [26] is not considered here for sake of simplicity). Moreover, Europeans are supposed to preferentially communicate with Free Blacks and to have only few direct interactions with Bozal Slaves. In practice, Europeans speak to Bozals only with probability *ε* in order to have a realistic minimal interaction, so that, when *ε* is negligibly small, Mulattos are the only mediators for the main (indirect) interaction between Europeans and Bozals. Further, a main factor for the development of creole languages is the lack of a common substrate language spoken by the African slaves. Bozal slaves were speaking different African languages; thus they were not able to communicate with each other in their mother tongues. We model this situation in a coarse fashion by stipulating that two Bozal slaves speaking in an African language *A* have only a probability *δ* to understand each other. Lastly, with a probability *γ*, individuals that are familiar with both the languages *E* and *A*, can *invent* a new language, namely the creole *C*. The contact ecology used in the model is summarized in Fig 2 and below we report a complete description of the model.

### Detailed model definition

We consider a population of *N* individuals divided in three subpopulations: Free Whites (or Europeans, *Eu*), Free Blacks, i.e., mainly Creole Mulattos (*M*), and Bozal slaves (*B*). The total population size, as well as the relative population sizes of *Eu*, *M* and *B*, are kept constant over the whole interaction process: *N*_*Eu*_+*N*_*M*_+*N*_*B*_ = *N*. The initial conditions are such that the Europeans and the Mulattos speak *E* (the European lexifier), while the Bozals speak *A* (their African mother tongue). We consider the fact that the African slave population was societally multilingual in an average way by introducing a probability *δ* that two individuals speaking *A* will understand each other. The game evolves as follows: at each step of the dynamics, two individuals are randomly selected and one is assigned the roles of Speaker (S), the other the role of Hearer (H). S randomly selects a language in his repertoire, which at the beginning contains only one item, and speaks it to H: (a) if the hearer does not already possess the language of the utterance in her repertoire and therefore cannot make sense of it, she learns it by adding it to her repertoire; (b) if she has it, the communication is successful if the language is *E* or *C*, while the communication is successful only with probability *δ* if the language is *A*. In case of success, both S and H retain in their repertoire only the language of the utterance; in the opposite case, nothing happens. Since we are interested in the emergence of a new hybrid language, we introduce the additional rule by which if, after the interaction, H has in her repertoire both languages *A* and *E* (or the three languages *A*, *E* and *C*), with probability *γ*, she will retain only the new language *C* (creole). The contact ecology is modeled in the following way that: (i) Europeans can only act as S; (ii) Europeans interact with Bozals only with probability *ε* (as shown in Fig 2).

In the framework of our modeling scheme, we can study how the languages spoken by Mulattos and Bozal evolve in time, and which language will ultimately be adopted by the two populations. (Recall that Europeans will not change their language *E*.) We found that in all the situations either *E* or *C* dominates, while *A*—the substrate language—is never able to spread in the population, due to fractionalization among the African languages. (See the S1 Supporting Information for conditions under which A can dominate, when the same African language is shared by a sufficiently large part of the Bozal slave population.)

Note that here we look at the language at a coarse grained and extremely simplified level and we don’t make explicit reference to language components, viz., the lexicon, syntax or grammar. From this perspective our modeling scheme tries to capture the microscopic linguistic interactions into coarse-grained interactions at the population level. This does not mean that we overlook truly linguistic processes like lexical borrowings. We simply take them into account in the macroscopic rules of our scheme. The main ingredient of the model, i.e., the probability that an individual speaking languages *A* and *E* can end up with a brand new language *C*, is precisely trying to capture, in a very simple way, all the processes through which the contact between languages *A* and *E* possibly lead to a hybrid language *C* (which presumably borrowed some features from *A* and *E*).

**Fig 3:**
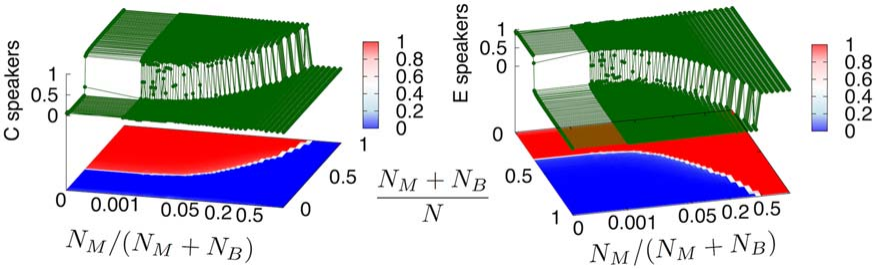
creole / non-creole transition. The two 3D graphs report the fraction of the Mulattos and Bozal populations speaking asymptotically *C* (**a**) and *E* (**b**), respectively, as a function of the coordinates *N*_*M*_/(*N*_*B*_+*N*_*M*_) and (*N*_*M*_+*N*_*B*_)/(*N*_*M*_+*N*_*B*_+*N*_*Eu*_). A relatively sharp transition line is observed separating the region where creole is predicted to emerge from the one in which the European language is predicted to dominate. Simulations are performed with parameters *γ* = 0.8, *ε* = 0.06, *δ* = 0.1 and *N* = *N*_*M*_+*N*_*B*_+*N*_*Eu*_ = 10000.

Our main results are summarized in Figs 1 and 3.

## Results

As explained above, States where a creole emerged and States where it did not can be clustered on the basis of census data alone. This is *per se* a first surprising result, given the complexity of the relevant population contact history. Moreover, this result allows to make predictions also at finer scales, discriminating regions within States where creole emergence is expected or not. This extension taps on the knowledge of finer demographic regional data within individual States. We will come back on this observation below and more extensively in the S1 Supporting Information.

In Fig 1 we show the prediction of our model: the separation line (black dashed line) predicted by the model divides the demographic space (represented in the plane $\frac{N_{M}}{N_{B}+N_{M}},\frac{N_{M}+N_{B}}{N_{M}+N_{B}+N_{Eu}}$) into two regions, where creole *C* (above the curve) and European *E* (below the curve) are respectively found to dominate. The black line clearly segregates the two regions of the experimental data. In order to clarify the meaning of the separation line predicted by the model, in Fig 3 we report the results of the model in a 3D plot. In particular, we report the fraction of the Mulattos and Bozal populations speaking *C* (Fig 3A) or *E* (Fig 3B) at the end of the dynamical process we modeled, as a function of the coordinates *N*_*M*_/(*N*_*B*_+*N*_*M*_) and (*N*_*M*_+*N*_*B*_)/(*N*_*M*_+*N*_*B*_+*N*_*Eu*_). Fig 3 show that the transition predicted from the region where *C* emerged to that where *E* spreads as the only language is a sharp transition, so that the probability with which the model can predict the emergence or not of a creole language is close to one for almost all the values of the demographic data. These results are quite surprising, given the relative simplicity of the modeling scheme adopted. They indicate that a suitable contact ecology can largely account for the creolization process, and that whether a creole language will emerge or whether the colonial European language will prevail without creolizing largely depends on the relevant population structure, in this particular case on the demographic distribution of White Europeans, Free Blacks or Bozal slaves within the total population.

**Fig 4:**
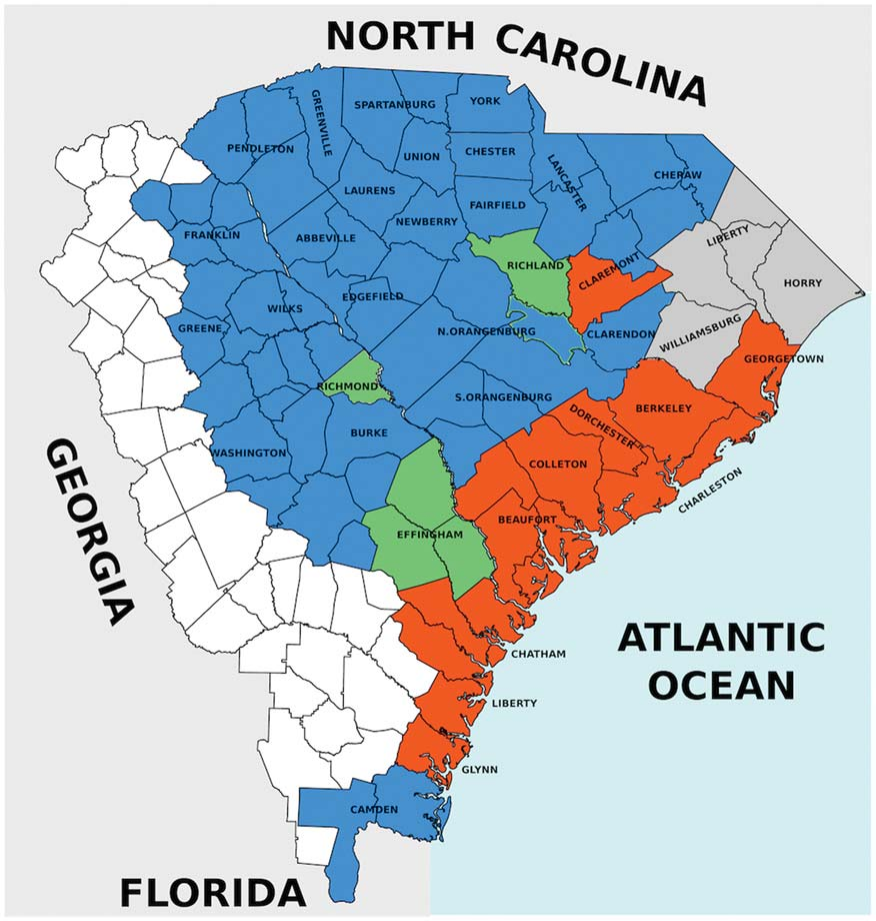
Detailed analysis of Georgia and South Carolina Counties. Prediction about the emergence of a creole language in those Counties and Parishes of Georgia (GE) and South Carolina (SC) for which census data collected in year 1790 are available. Blue Counties lie below the transition stripe shown in Fig 1 (see also Fig H and I in the S1 Supporting Information) so that our model predicts that creole did not emerge. On the contrary, orange Counties lie above the transition stripe where our model predicts the formation of creole. The green Counties lie in the grey transition region. Although modern County boundaries are shown, only the labels corresponding to the Counties existing in year 1790 are displayed. Since only part of Georgia was colonized in 1790, modern Counties that were not yet colonized are depicted in white (the western part of modern Georgia has been cut out). The dark gray Counties, though already existing in 1790, were not involved in the census operations. The boundary between GE and SC is marked by the river Savannah. This map was produced with the *Inkscape* open source software (*http://www.inkscape.org/*).

Let us now look more closely at the results presented so far. Already in Fig 1 we can note that the discrimination between States where a creole emerged and States where it did not is not clearcut near the transition line. In order to clarify the meaning of the closeness to the transition line, let us focus on those specific States for which the distinction creole vs. non-creole is uncertain, i.e., those close to the transition line like South Carolina, Georgia, Alabama, Virginia, Mississippi and Louisiana. A first important observation concerns the population distribution inside each State. For the analysis performed so far we always considered the population division into Free Whites, Free Blacks and Bozal slaves at the level of the whole State, though strong heterogeneities could be present. This is the case, for instance, in Georgia, South Carolina and Virginia. Most of the slave populations in these States were concentrated on the coast, which was avoided by Whites who could afford to live elsewhere. While the proportion of slaves relative to that of Whites constitutes 9 to 1 in the coastal area in places such as South Carolina [27], the slaves were in the minority on the cotton plantations, where they hardly exceeded 40% of the population, and even a smaller proportion on the farm [28]. Consequently, Gullah, the English-based creole of Georgia and South Carolina, emerged only in the coastal areas. No Gullah(-like creole) emerged in the State’s hinterland. Our modeling scheme confirms this scenario since the counties where a creole emerged are correctly located well above the transition lines. Fig 4 illustrates the model’s predictions for Georgia and South Carolina when a finer population structure at the level of counties is considered (see S1 Supporting Information for the tables reporting the population structure of those States at the county level). Note that once all the parameters are fixed, the predictions of our model solely depend on the demographic composition of the considered region, where the relevant observables are percentages and not absolute values. This allows our modeling technique to detect a non-uniform behavior within individual States, in the above considered case confirming the likelihood of the emergence of the Gullah only in the coastal areas. The same scenario holds in Virginia (see S1 Supporting Information for details), where the population composition on the coastal marshes was similar to that in coastal South Carolina and the local African American Vernacular English (AAVE) variety that developed there has been reported to sound Gullah-like. These results are very encouraging since they prove that our modeling scheme can be modulated in order to account for local specificities in the emergence of creoles. We refer the reader to the S1 Supporting Information for a detailed discussion of the results for individual States.

Another interesting observation concerns the meaning of the transition line observed in Fig 1. The transition line was obtained with a unique set of values of the model parameters in order to maximize the overall prediction power of the modeling scheme. This implies that specific sets of values could better describe local social structures in different States. In the S1 Supporting Information we discuss the dependence of this line on the model parameters. It turns out that the the only parameter that sensibly shifts the transition line is the parameter *ε*, which mimics the strength of the segregation between Europeans and Slaves. From this perspective it is worthwhile remarking that the prediction for specific States could be improved by tuning, in a finer and controlled way, the value of the segregation parameter *ε*. For instance, cotton plantations were smaller and required fewer laborers than rice fields and sugarcane plantations. This in turn corresponds to a different segregation regime, viz., higher in sugarcane plantations and rice-fields than in cotton plantations, where it was institutionalized much later in the history of the United States. This variation helps us explain why similar global demographic percentages can still lead to different outcomes regarding the emergence of creoles, depending on the specificity and strength of the segregation regime. This can explain for instance the lack of a creole in Mississippi. During the 18th century, Mississippi was a French colony, sparsely populated, and its main economy was based on fur trade. Its shift to agricultural economy, after being incorporated in the United States in 1817, was marked by the development of cotton plantations. Similar arguments could be made for Alabama and for Louisiana (whose history of double colonization, first by the French and later by the Americans, makes the evolutionary picture more complex). A detailed discussion of the model’s results for individual States is provided in the S1 Supporting Information.

## Discussion

In summary, we introduced a modeling scheme to investigate the emergence of creole languages in ecologies where language mixing occurred through the interactions of colonizers with slaves, and among the latter, with the Mulattoes/Creoles typically playing an important role in seasoning the Bozal slaves. The model predicts that the relevant parameters to account for the emergence of a creole, as opposed to the diffusion of the colonizing language, include the relative proportions of Europeans, Free Colored and Blacks. We informed our modeling scheme with census data from the USA and the Caribbean; and we were able to make fairly accurate predictions regarding whether a creole emerged in a particular contact ecology. In addition, the model allowed us to identify borderline situations where more detailed historical and linguistic analyses could be focused. Thanks to its flexibility, we believe our modeling scheme could become an important tool of investigation for scholars interested in the emergence of contact languages. Our modeling scheme can be easily modified and adapted to very different contact ecologies by varying: (i) the spatial resolution, to investigate how local census data affect the subsequent linguistic development and evolution; and (ii) the time resolution, to investigate the temporal conditions that favor or disfavor the emergence of creole or other vernaculars. In addition, the values of the parameters in our modeling scheme can be easily fine-tuned to different contact ecologies to identify places that call for a historical explanation or where different competing hypotheses could be challenged using actual data. Finally the modeling scheme proposed here can be generally applied to many contact phenomena in different cultural domains [1–3, 5], like for instance the emergence of dialects, the competition among different languages, the hybridization processes like those undergone by languages with an important number of non- native speakers and more generally all processes where different cultural features come in contact.

## Supporting Information

S1 Supporting Information Pdf file including 10 figures and 8 tables. (PDF)
