# Supplementary material for: Modeling the Emergence of Contact Languages
Source: PLoS One. 2015 Apr 15;10(4):e0120771. doi: 10.1371/journal.pone.0120771 (PMC4398412; doi:10.1371/journal.pone.0120771)
Supplement: S1 Supporting Information — (PDF) [file pone.0120771.s001.pdf]

Modeling the emergence of contact languages

Supporting Information

Francesca Tria[1], Vito D. P. Servedio[2,3],
Salikoko S. Mufwene[4] & Vittorio Loreto[3,1,5]

April 2, 2015

[1] Institute for Scientific Interchange (ISI), Via Alassio 11/c, 10126 Torino, Italy
[2] Institute for Complex Systems (ISC-CNR), Via dei Taurini 19, 00185 Roma, Italy
[3] Sapienza University of Rome, Physics Dept., Piazzale Aldo Moro 2, 00185 Roma, Italy
[4] Univ. of Chicago, Dept. of Linguistics, 1010 E. 59th Street, Chicago, IL 60637, USA
[5] SONY-CSL, 5, Rue Amyot, 75005, Paris, France

1 Analysis of the model parameters

In this article we study the dependence of the model predictions on the parameters ϵ, γ and δ. We found that the segregation regime, parametrized by ϵ, plays an important role in the emergence of creoles languages. In particular, we found that for States near the line separating the two regions where Creoles emerged or did not, the model predictions vary accordingly to the hypothesized strength of the segregation regime. Higher contact between Europeans and Bozal Slaves disfavor the emergence of a creole language, favoring the acquisition of the European lexifier by Mulattos and Bozal Slaves (see Fig. A in this S1 file).

The transition line between the region where Creole or European dominate is conversely left unaltered by changing the parameter δ, representing the level of multilingualism of African languages (see Fig. B in this S1 file). However, when a single African language A is hypothesized to be understood by the majority of African slaves, a second transition appears, dividing a region into where the model predicts the emergence of creoles and a region where the model predicts that the African language A is adopted by Mulattos an Bozal slaves (see Fig. B in this S1 file).

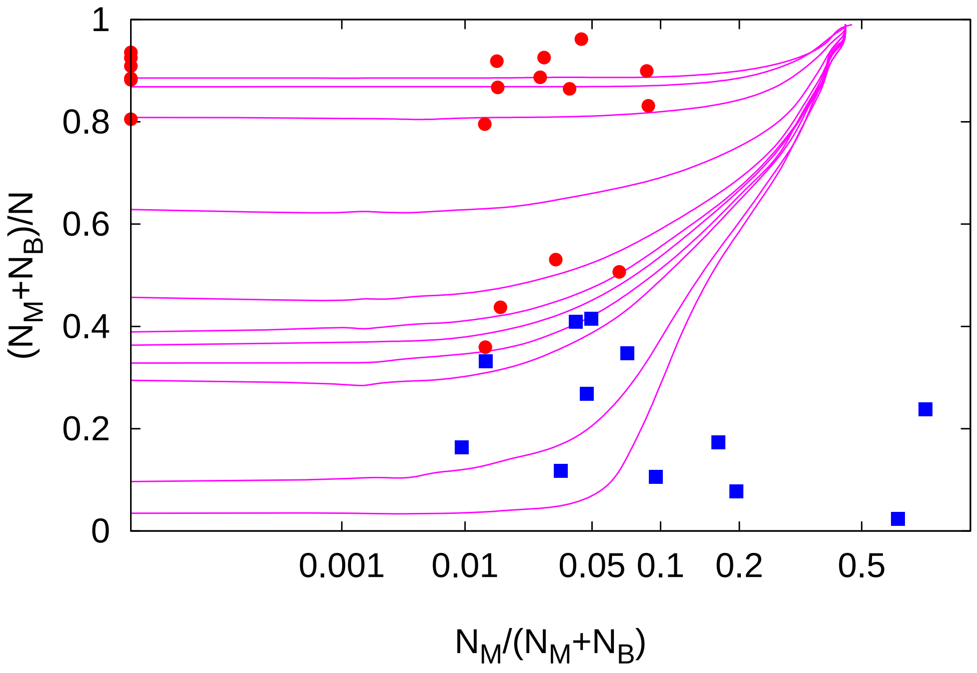

Figure A: **Dependence of the model predictions on the probability ϵ of interaction between Europeans and Bozal slaves.** We report the division line between a region where the model predicts creole emergence (above the line) and where the model predicts no creole emergence and E as the unique shared language (below the line). The different lines are for the values of ϵ, from bottom to top: 0.01,0.02,0.05,0.06,0.07,0.08,0.1,0.2,0.5,0.8,1.0. All the other parameters of the model are set as in the main text: $\gamma = 0.8$ and $\delta = 0.1$. Red points indicate States where Creole did emerge, while blue squares indicate States where Creole did not emerge (see also the main text).

However this latter situation does not represent the historical reality in the colonies (please also see the discussion in the next section). Finally, the actual value of the γ parameter, provided that it is not too low, does not affect the model predictions (see Fig. C in this S1 file). In sum, while the model shows some dependence on the two parameters related to the specific contact ecology considered, thus allowing for more thorough investigation of particular historical situations, its predictions are largely stable against the choice of the model's parameters.

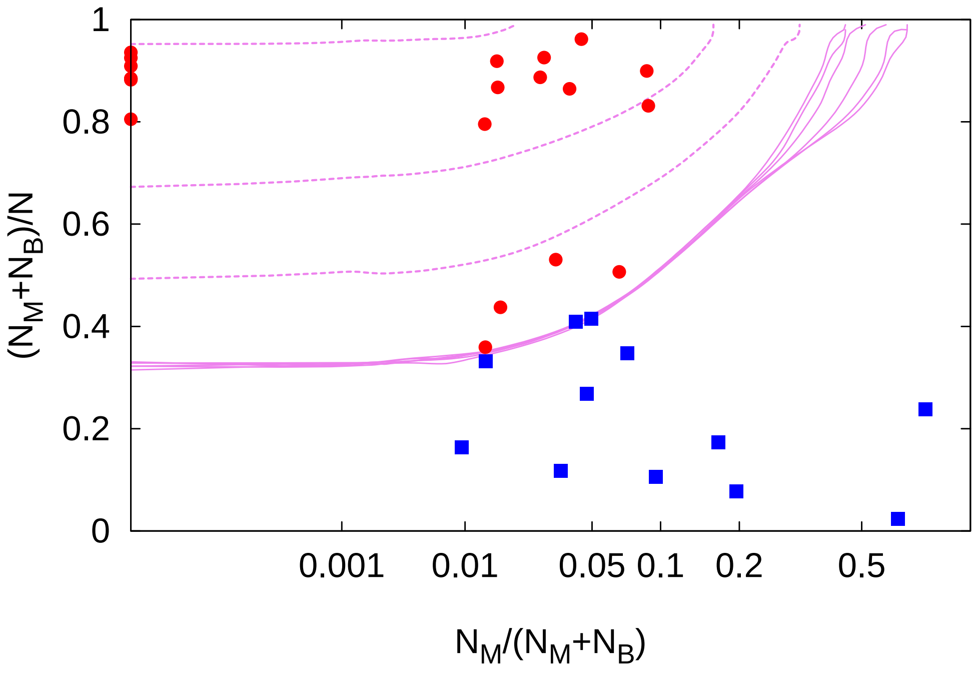

Figure B: **Dependence of the model predictions on the probability δ that two individuals speaking A will understand each other.** We report the results for the values of δ: 0.05,0.1,0.2,0.5,0.8,1.0. Here, for some values of δ, we observe two different transitions lines. Common to all the δ-values is the transition from a region where Creole emerged (above the solid line) and where E remains as the unique spoken language (below the solid line). Further, for the higher values of δ, we observe a second transition, from the region where Creole emerged (below the dashed line), and a region where A is retained as the language of Mulattos and Bozal slaves (above the dashed line). Dashed lines correspond to the δ values, from top to bottom: 0.5,0.8,1.0. Again, red points indicate States where Creole did emerge, while blue squares indicate States where Creole did not emerge. The other parameters values are $\epsilon = 0.06$ and $\delta = 0.1$.

2 Robustness of the model's predictions

For the sake of completeness, we report here the predictions of our modeling scheme for different values of the model parameters, when taking as initial con-

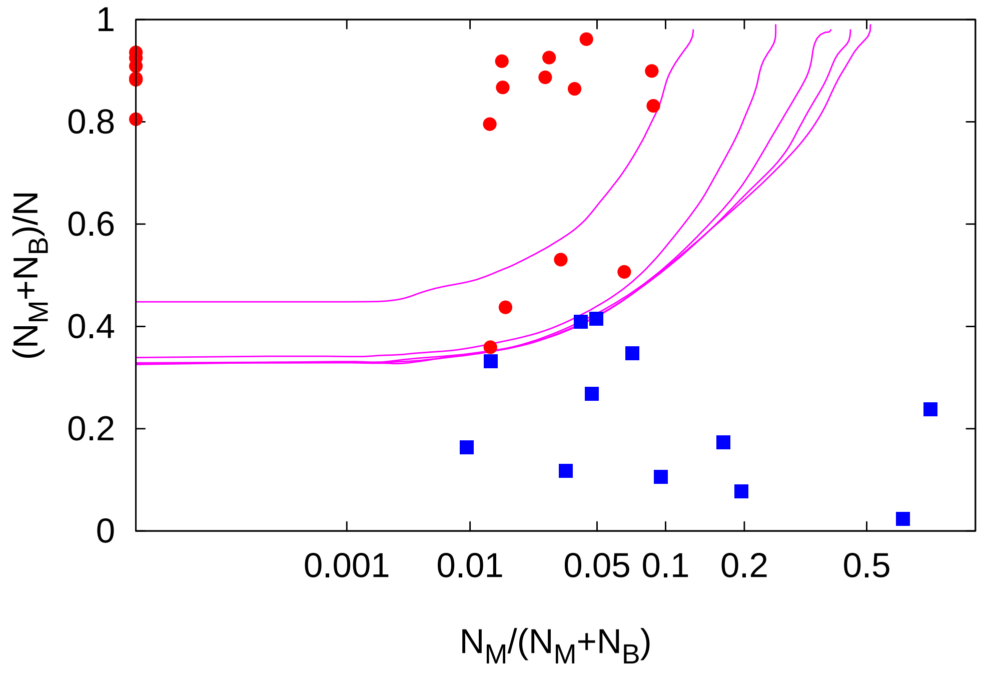

Figure C: **Dependence of the model's predictions on the probability γ of inventing a new language C out of A and E.** We report the division line between a region where the model predicts creole emergence (above the line) and where the model predicts no creole emergence and E as the unique shared language (below the line). The different lines are for the values of γ, from top to bottom: 0.2,0.4,0.6,0.8,1.0. The other parameters of the model are set as in the main text: $\epsilon = 0.06$ and $\delta = 0.1$. Red points indicate States where Creole did emerge, while blue squares indicate States where Creole did not emerge.

ditions the actual population composition of each individual State, as reported in the census data discussed in the main text. In particular, we performed a set of stochastic simulations with the following set of parameters: $\gamma \in \{0.3, 0.6, 0.8, 1.0\}$; $\epsilon \in \{0.00, 0.01, 0.05, 0.10, 0.20, 0.40\}$; $\delta \in \{0.1, 0.2, 0.4\}$ for a total of 96 simulations per State. In addition, since the initial date of the creolization process cannot be fixed with fine precision, we studied different initial conditions when census data at different years were available. Fig. D in this S1 file illustrates the predictions of our modeling scheme for the States considered. The idea is that

the creolization in those States that lie at the proximity of the transition band of Fig.1 of the main text should be more susceptible to parameter variability and they deserve a more careful analysis that also takes into account their particular population composition as well as other historical elements. It is important to remark how the transition band of Fig. 1 of the main text has been drawn using the same set of parameter values, while it is reasonable to imagine that not all the States are well represented by the same set of parameters. The next section is devoted to a more detailed analysis of individual States.

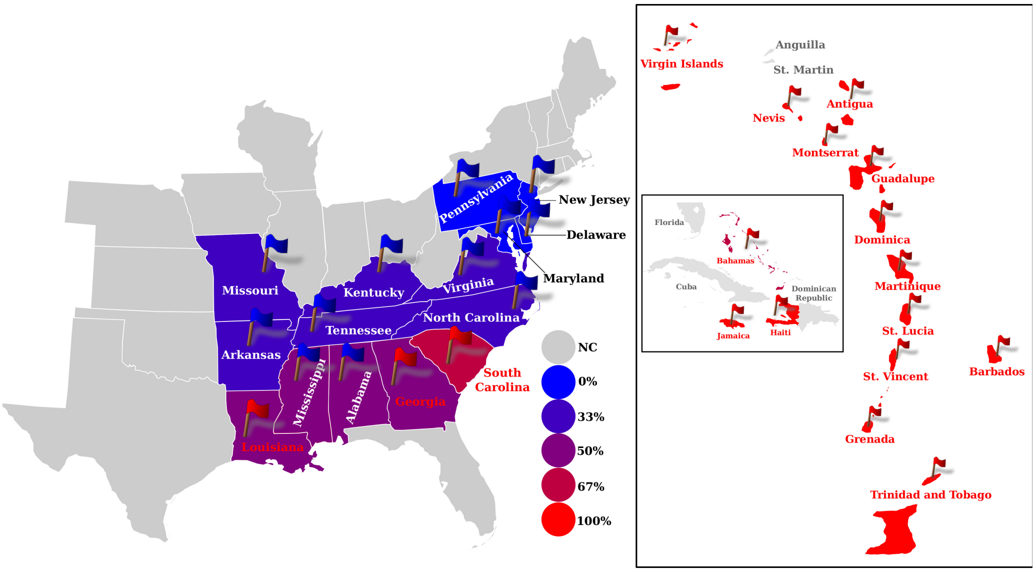

Figure D: **Prediction of the emergence of Creole in US states and the Caribbean.** The map reports, for each US State investigated, the comparison between the model's prediction of the emergence or non-emergence of Creole and the actual situation. The likelihood of the emergence of Creole is encoded in the color each state is represented with, by using the color code reported in the legend. The flag colors mark whether a Creole language emerged (red flag) or not (blue flag), while States are colored according to the likelihood of Creole emergence as predicted by the model. This map was produced with the *Inkscape* open source software (*http://www.inkscape.org/*).

3 The model's results for individual States

In this section we go deeper in analyzing the model's predictions in individual States. For the sake of clarity we display again here Fig. 1 of the main text as Fig. E in this S1 file.

Creolization in the States that lie at the border of the two regions, e.g., Alabama (1820), Louisiana (1850), Georgia (1790), Virginia (1790), Mississippi (1800), appears to be more dependent upon the choice of parameters than, for example, Barbados (1786) and Missouri (1810), which in contrast were found to be more stable and lie far from the transition line. More explicitly, no particular parameter combination suggests linguistic creolization in Missouri (1810). This is a consequence of the fact that its colonial population had a White majority. Unlike in coastal South Carolina, where race segregation was introduced early in the 18th century, the Missouri population was not racially segregated until the late 19th century, when the Jim Crow laws were institutionalized. Creolization has typically been associated with large plantation settlement colonies on which the African slaves were the overwhelming majority early in the colonial history and race segregation was introduced concurrently [1, 2, 3, 4, 5].

All parameter combinations except those with $\delta > 0.8$ suggest creolization development in Barbados (1786). According to our model, if we let the Bozals of Barbados speak the same African language ($\delta = 1$), they would preserve it despite the pressure to adopt the European language and creolize. This special situation of Bozals speaking the same African idiom has never been reported for any colonies. Instead, there were cases where populations from particular parts of Africa represented an important part of the slave population at a particular time, and they influenced the way the Creole developed, e.g. Berbice Dutch in Guyana, Sranan and Saramaccan in Surinam, Haitian Creole (Saint Domingue), and perhaps Jamaican Creole [6, 7, 8, 9]. In all cases, we should bear in mind that societal multilingualism among African slaves generally disfavored the retention of a substrate language as a vernacular, especially in ecologies were race segregation was institutionalized later in their history, in the late 19th century, as in the cotton- and tobacco-plantation States. Thus, Alabama's Bozals were ecologically driven towards adopting the dominant European language. More generally, in the States with a high Black dominance our model with $\delta \simeq 1$ predicts that the Bozals' African language would prevail, suggesting that the heterogeneity of African languages among Bozals may have played a major role in the process of creolization.

Let us now examine specific examples more closely.

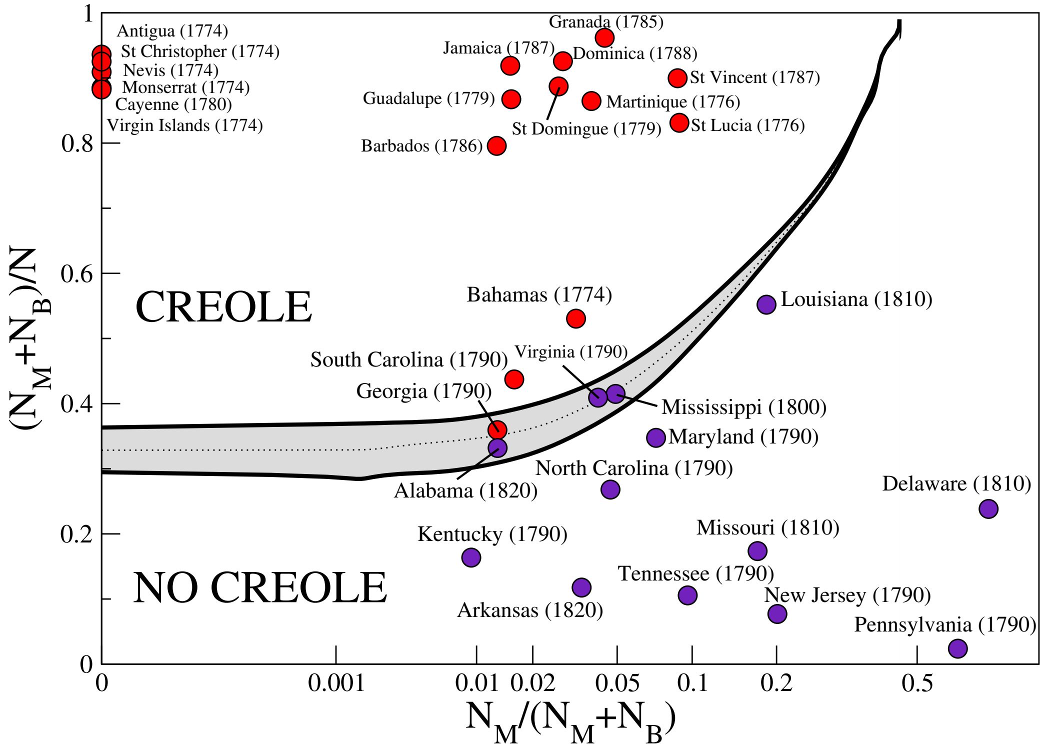

Figure E: **Clustering in the Creole formation process.** As in Fig. 1 of main text: Points are the projection of the census data (See Tables A-D in this Supporting Information S1) in the plane $(N_M/(N_B+N_M),\ (N_M+N_B)/(N_M+N_B+N_{Eu}))$. Red circles mark counties where a Creole language emerged while purple ones identify States where a Creole language historically did not emerge. The gray strip is the outcome of our modeling scheme and separates the regions where respectively Creole C (above the strip) and European E (below the strip) represent the dominant language (i.e., spread among more than the 80% of the population) in the Mulattos and Bozal populations in the asymptotic states of the model. The two black curves delimiting the gray stripe and the dashed line in the middle are obtained by simulations performed with the same parameters $\gamma = 0.8$, $\delta = 0.1$ and $N = N_M + N_B + N_{Eu} = 10000$, with ϵ ranging from 0.05 (bottom black curve) to 0.07 (upper black curve), passing through 0.06 (dashed curve). The horizontal axis has been artificially expanded by a power 0.2.

Virginia The population distribution was very uneven in Virginia at the beginning of the 19th century. Fig. F in this S1 file reports the projection of the census data. It suggests that coastal counties (green circles) display a larger likelihood for the emergence of a creole language. To date nobody has suggested that a creole developed in the area. However, it was reported to Salikoko Mufwene in the late 1980s that African Americans in rural and marshy coastal Virginia speak a variety of English different from African American English (AAVE) spoken in the hinterland and closer to Gullah. Future field research will shed light on this question.

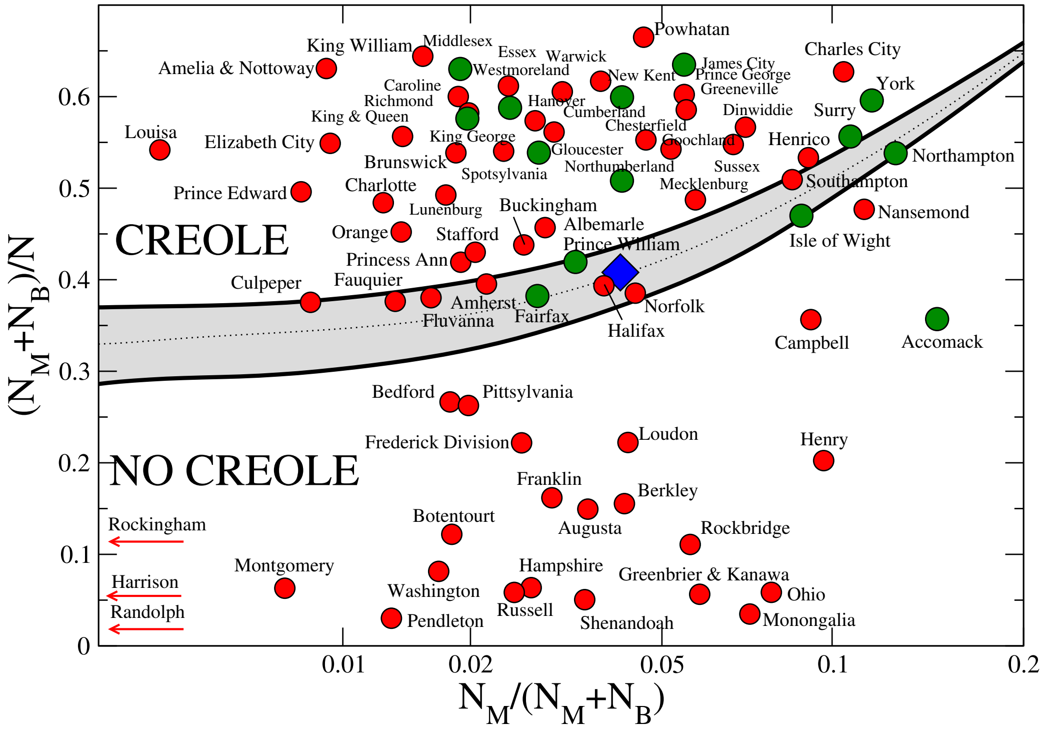

Figure F: **Detailed analysis of Virginia counties in 1790.** Points are the projection of the census data (See Table E of this S1 file) in the plane $(N_M/(N_B + N_M),\ (N_M + N_B)/(N_M + N_B + N_{Eu}))$. Green and red circles mark counties lying on the coast and in the hinterland, respectively. The large blue diamond refers to the Virginia State as a whole. The three counties at the bottom left lie at a zero x value. The gray stripe and axes definitions are the same of Fig. E of this S1 file.

Fig. G in this S1 file illustrates the model's predictions for the emergence of a

creole language in Virginia's counties and parishes of 1790.

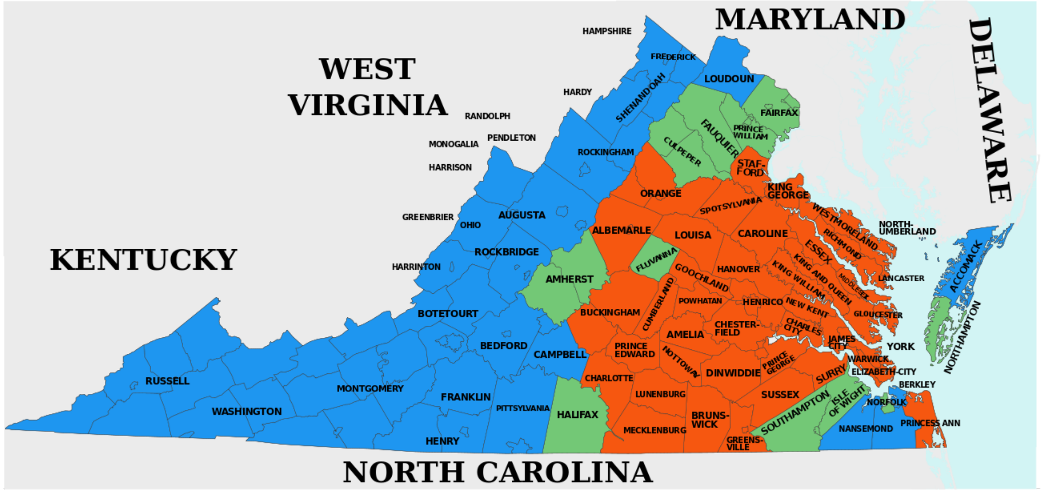

Figure G: **Detailed analysis of Virginia counties.** Here we report the predictions about the emergence of a creole language in those counties and parishes of Virginia for which census data collected in year 1790 are available. The blue counties lie below the transition stripe shown in Fig. F of this S1 file so that our model predicts that Creole did not emerge. On the other hand, orange counties lie above the transition stripe where our model predicts the formation of Creole. The green counties lie in the gray transition region. Although modern county boundaries are shown, only the labels corresponding to the counties existing in year 1790 are displayed. This map was produced with the *Inkscape* open source software (*http://www.inkscape.org/*).

South Carolina and Georgia The population distribution was very uneven also in South Carolina and Georgia. Fig. H and I in this S1 file report the census, county by county in those two states. They make evident the strong heterogeneity in the relative populations of Whites, Free Blacks and Bozal Blacks across the different counties. At the same time a larger likelihood for the emergence of a creole language is observed in coastal counties, where Gullah emerged. A summary of the model's predictions for the emergence of a creole language in South-Carolina and Georgia counties of 1790 were already presented in Fig. 4 of the main text.

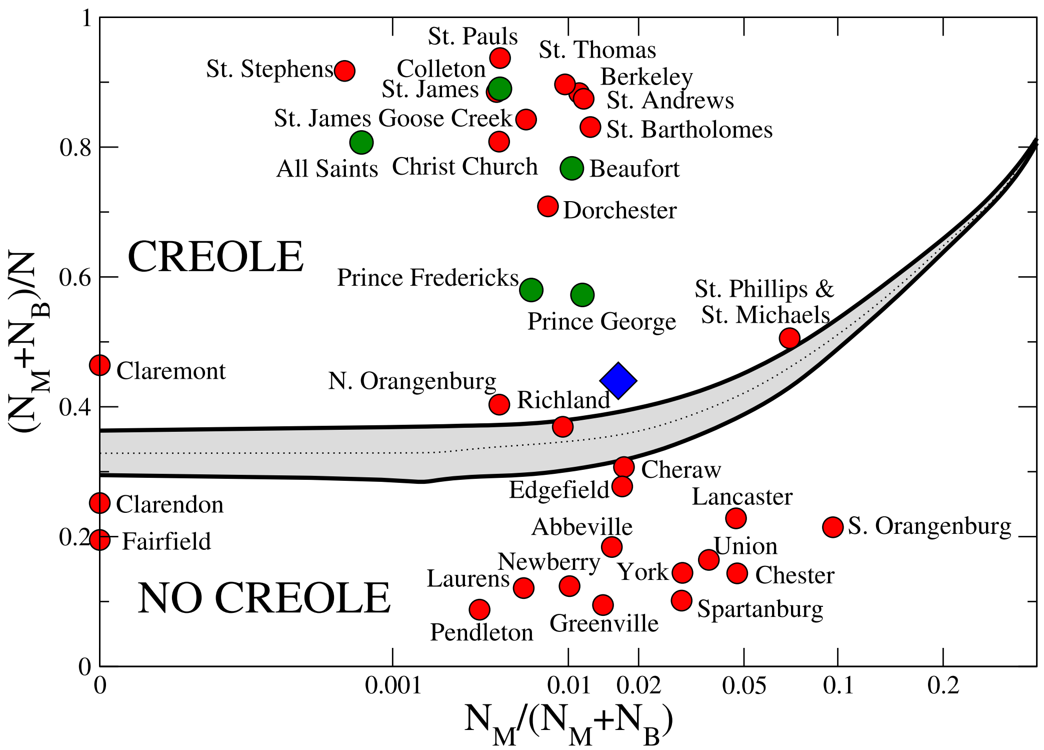

Figure H: **Detailed analysis of South Carolina counties in 1790.** Points are the projection of the census data (See Table H in this S1 file) as in Fig. F of this S1 file, the large blue diamond referring to the South Carolina State as a whole. Green and red circles mark counties lying on the coast and in the hinterland, respectively. The gray stripe and axes definitions are the same of Fig. E of this S1 file.

Lousiana Louisiana represents an interesting case since we have access to census data regarding several decades, namely, 1810-1820-1830-1840-1850. In Fig. J (top) of this S1 file we display those data and it turns out that the likelihood for the emergence of the Creole seems to increase with time, i.e., the relative population sizes of Whites, Free Blacks and Bozal Blacks of 1850 lies above the transition line while in 1810 it lies below. We speculate that the lack of census data about 18th century, when Louisiana was a French colony, distorts the fact that large French sugarcane plantations existed then that produced a French creole. Also, although the 19th-century census figures indicate a population increase, correlated largely with the introduction of the cotton industry to the State, then part

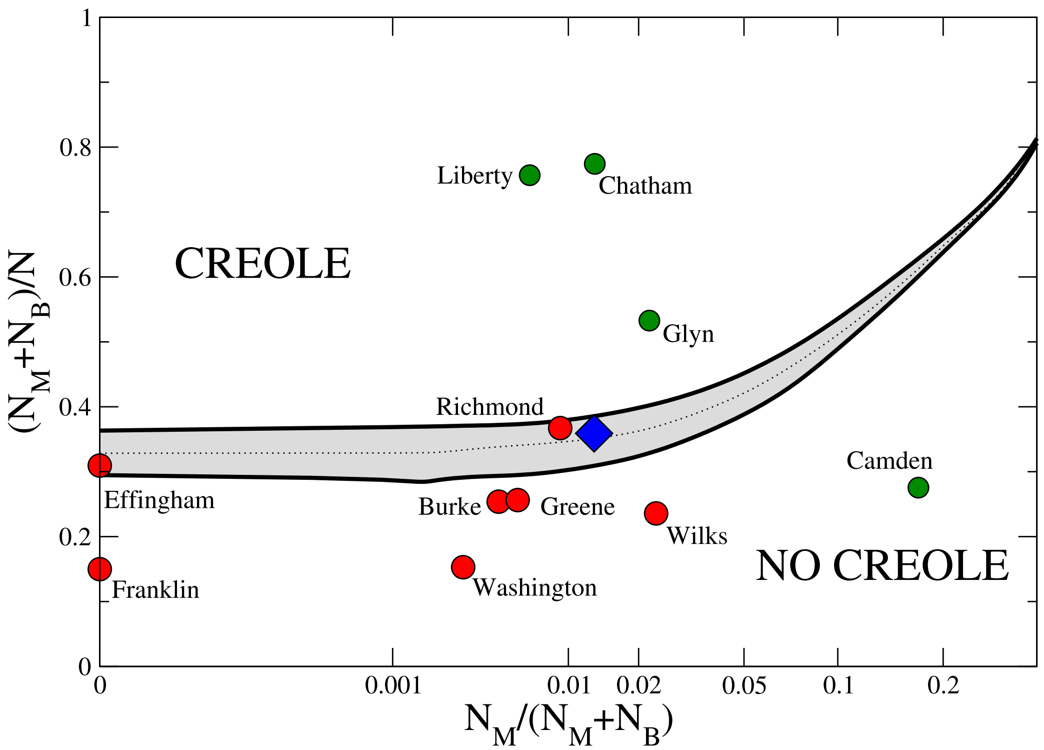

Figure I: **Detailed analysis of Georgia counties in 1790.** The points are the projection of the census data (See Table G in this S1 file) as in Fig. F of this S1 file, the large blue diamond referring to the Georgia State as a whole. Green and red circles mark counties lying on the coast and in the hinterland, respectively. The gray stripe and axes definitions are the same of Fig. E of this S1 file.

of the expanding United States, they do not point out an important difference in population structure. The cotton plantations were smaller in size and less densely populated compared to sugarcane plantations and rice fields (in the case of South Carolina and Georgia); generally they produced closer approximations of the European language, AAVE in the United States. We should also keep in mind that there were officially no more Bozal slaves after the abolition of the slave trade in the early 19th century and certainly not after the abolition of slavery in the mid-19th century. The slaves that came to the cotton plantations of Louisiana came from other places in the United States, especially Virginia, which made a lot of money from the domestic slave trade. So, these were not Bozal slaves. They came

to Louisiana already speaking an ancestor of AAVE and there was thus no reason for a creole to develop.

This example is very interesting because it demonstrates how our modeling scheme could turn out to be a valid tool to support field works in studies of creolization. For instance different predictions can be tested depending on the demographic composition of the population at the time Creole formation is expected. This implies that before getting to firm conclusions one has to carefully match the modeling scheme with the local historical conditions in order to obtain reasonable estimates of the parameters' values to be used in the model simulations.

Alabama A similar argument could be repeated as for Lousiana. The model predicts a likelihood of 50% of Creole emergence in Alabama (see Figs. E and J (top) in this S1 file), where in reality no English creole emerged. This suggests that direct interactions between slaves and Europeans might have been stronger in Alabama (1820) than in Lousiana (1850). The colonial history of the region actually explains this. Although both States were originally part of the much larger French colony of Louisiana up to the late 18th century, Alabama, which had not launched in the sugarcane industry in the 18th century, would develop in the 19th century primarily on the cotton cultivation economy, while Louisiana would continue its sugarcane cultivation until the middle of the 19th century, before letting the cotton industry prevail afterwards. Unlike sugarcane plantations, which had slave-majority laborers, the cotton plantations were smaller, as explained above, and depended on white-majority laborers. As explained in Mufwene [10, 5], cotton plantations in the United States produced no creoles, unlike rice fields in coastal South Carolina and Georgia, and the sugarcane plantations of the Caribbean. Unsurprisingly, no English creoles emerged in Alabama and Louisiana. That is, cotton cultivation in the United States, associated with English, produced no Creole, whereas sugarcane cultivation, associated with the French colonization and thriving before the Louisiana Purchase (1803), produced a French Creole. We must remember in the particular case of Louisiana that two different economic cultures coexisted and competed with each other for a while in the history of this State; and affected the evolution of the two colonial languages, English and French, in different ways.

Mississippi In Mississippi too, while eventually no Creole developed, the present model suggests that the likelihood of its formation grows in time (Fig. J (top) in this S1 file). Comparing the census data of Mississippi with those of South Car-

olina (as both have comparable black majorities after 1840), one may wonder why Mississippi did not produce a Creole, while South Carolina did. There are again many reasons for this differential evolution. Coastal South Carolina had Black majority in the early 18th century already (1720-1740) [11]. Although the proportions would shift gradually in the 19th century, coastal South Carolina maintained its Black majority until the mid-20th century and has maintained Gullah to date, the English creole that developed in its rice fields in the 18th century. During the 18th century, Mississippi was a French colony, sparsely populated; and its main economy was fur trade. When it shifted to agricultural economy, after being incorporated in the United States in 1817, it developed cotton plantations, which were smaller and required fewer laborers than rice fields and sugarcane plantations, as explained above. (Many of its slaves also came from cotton and tobacco plantation States, including Virginia, which produced no creoles either.)

Regarding its demographics, Mississippi became comparable to Virginia, with its tobacco plantations. The higher proportion of Blacks in Mississippi, concentrated along rivers and in the Delta area, is comparable to that of Virginia in the orange-shaded areas in the map of Fig. G in this S1 file, where more basilectal Gullah-like varieties of AAVE (in the opinion of some native speakers) appear to have developed. Note also that overall, South Carolina was more densely populated than Mississippi. Although the proportion of South Carolina's slave population kept decreasing in the 19th century, the slave population remained the overwhelming majority in the coastal areas, because Whites also avoided them, as they consisted of marshes and were infested with malaria. Race segregation was also institutionalized earlier in coastal South Carolina, in 1720, unlike in Mississippi, in the late 19th century. According to data cited by Wikipedia (checked on December 24th, 2013), the Mississippi enslaved population comprised emph436,631 in 1860, or 55% of the State's total of 791,305. Typically, the territories that developed a Creole reached the slave majority early in the histories of the colonies and implemented race segregation concurrently!

There are indeed several other parameters that modelings of differential language evolution will have to factor in, although ours, based on census data, appears to have approximated that history quite well. Our scheme has the merit of raising pertinent issues that ask for the kinds of explanations that we have also provided in our narrative. We have generally learned that demographics applied indiscriminately to overall States or colonies sometimes lead to inaccurate predictions of where we can expect a Creole to have emerged. Indeed, where the modeling predictions and reality do not match, we are prompted to look into history to explain these discrepancies.

References

[1] Mufwene SS (2001) The Ecology of Language Evolution. Cambridge: Cambridge University Press.

[2] Mufwene SS (2005) Créoles, écologie sociale, évolution linguistique. Paris: L'Harmattan.

[3] Mufwene SS (2008) Language Evolution Contact, Competition and Change. Continuum.

[4] Schneider EW (1995) Black-white language contact through the centuries: Diachronic aspects of linguistic convergence or divergence in the united states of america. In: Fisiak J, editor, Linguistic Change under Contact Conditions, Berlin: Mouton de Gruyter. pp. 237–252.

[5] Mufwene SS ((to appear)) The english origins of african american vernacular english: What edgar schneider has taught us. In: Hoffmann T, Kauzsch A, Huber M, Buschfeld S, editors, Festschrift to Edgar W. Schneider, Elsevier.

[6] Aboh E (2006) Complementiation in saramaccan and gungbe: the case of c-type modal particles. Natural language and linguistic theory 24: 1–55.

[7] Lefebvre C (1998) Creole genesis and the acquisition of grammar: The case of Haitian creole. Cambridge: Cambridge University Press.

[8] Kouwenberg S (1994) A Grammar of Berbice Dutch Creole. Berlin: Mouton de Gruyter.

[9] Alleyne MC (1996) Syntaxe historique créole. Hommes et societes. Paris: Editions Karthala.

[10] Mufwene SS (2009) The indigenization of english in north america. In: Hoffmann T, Siebers L, editors, World Englishes - Problems, Properties, Prospects: Selected Papers from the 13th IAWE Conference. Amsterdam: Benjamins, pp. 353–368.

[11] Wood PH (1974) Black Majority: Negroes in Colonial South Carolina from 1670 through the Stono Rebellion. New York: Knopf.

	Year	Europeans	Free Colored	Blacks	Creole	European
Georgia	1790	52886	398	29264	50%	50%
	1800	101678	1019	59404	50%	50%
	1810	145414	1801	105218	50%	50%
	1820	189566	1763	149654	50%	50%
	1830	296806	2486	217531	50%	50%
	1840	407695	2753	289944	50%	50%
	1850	521572	2931	381682	50%	50%
Louisiana	1810	34311	7585	34660	25%	75%
	1820	73383	10476	69064	29%	71%
	1830	89441	16710	109588	44%	56%
	1840	158457	25502	168452	44%	56%
	1850	255491	17462	244809	50%	50%
South Carolina	1790	140178	1801	107094	50%	50%
	1800	196255	3185	146151	50%	50%
	1810	214196	4554	196365	63%	38%
	1820	237440	6826	258475	67%	33%
	1830	257863	7921	315401	67%	33%
	1840	259084	8276	327038	67%	33%
	1850	274563	8960	384984	67%	33%

Table A: Census data relative to the populations of Europeans (Whites), Free Colored (Mulattos) and Blacks (Bozals) in those states that eventually developed the creole language. The last two columns show the result of the model stochastic simulation with the set of parameters described in the text. In the evaluation of the percentage figures the cases with $\delta = 1$ (Bozals speaking the same African language) were left out since they all resulted in the adoption of the language B as their vernacular. The single simulation is never ambiguous: either the whole (almost) Mulatto and Bozal populations end up with the Creole language or with the European, or with the African one in case $\delta = 1$.

	Year	Europeans	Free Colored	Blacks	Creole	European
Antigua	1774	2590	0	37808	100%	0%
Bahamas	1774	2052	77	2241	64%	36%
Barbados	1786	16167	838	62115	100%	0%
Cayenne	1780	1385	0	10539	100%	0%
Dominica	1788	1236	445	14967	100%	0%
Granada	1785	996	1115	23926	100%	0%
Guadalupe	1779	13261	1382	85327	100%	0%
Jamaica	1787	23000	4093	256000	100%	0%
Martinique	1776	11619	2892	71286	100%	0%
Monserrat	1774	1300	0	10000	100%	0%
Nevis	1774	1000	0	10000	100%	0%
St Christopher	1774	1900	0	23462	100%	0%
St Domingue	1779	32650	7055	249098	100%	0%
St Lucia	1776	2397	1050	10752	97%	3%
St Vincent	1787	1450	1138	11853	100%	0%
Tobago	1776	2397	1050	10752	97%	3%
Virgin Islands	1774	1200	0	9000	100%	0%

Table B: Same as Table A of this S1 file.

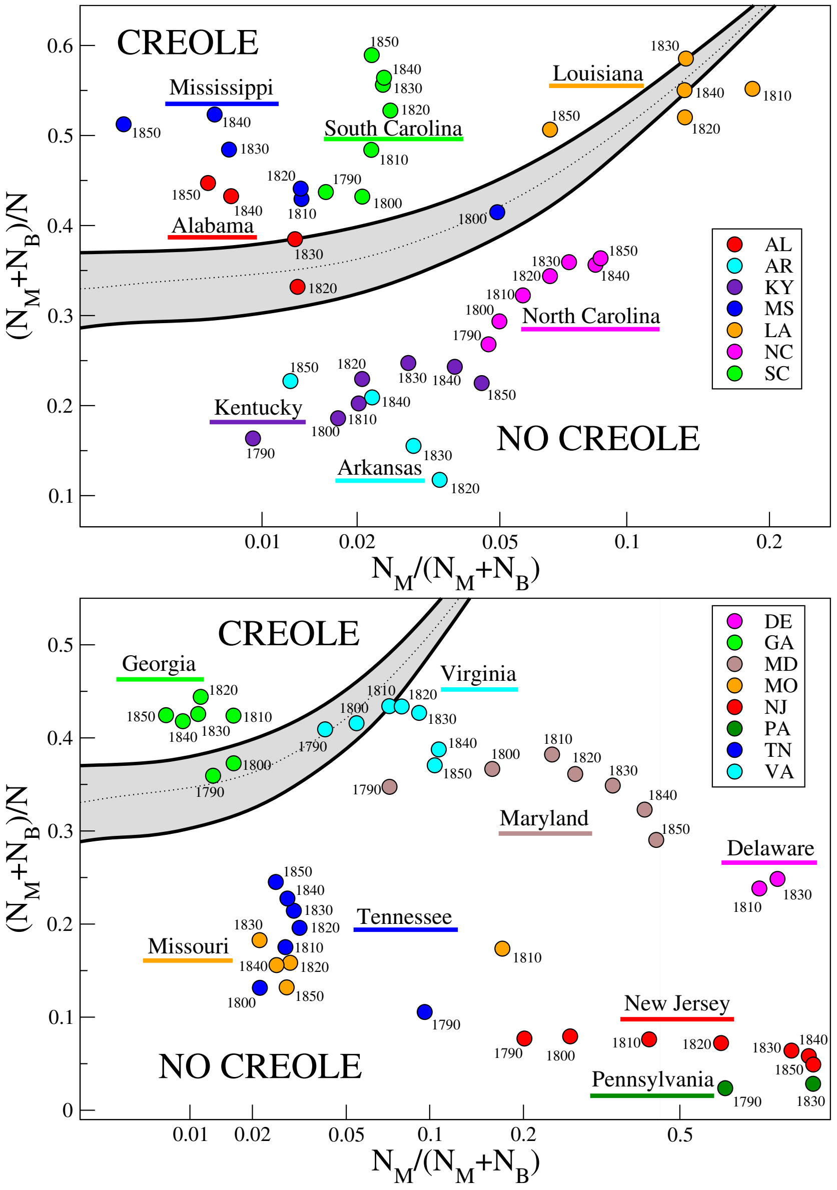

Figure J: **Clustering in the Creole formation process for census data in different decades.** The plot is separated in two figures for the sake of clarity. Both contain the same information for different States: Points are the projection of the census data (See Tables A-D in this S1 file) for different year decades in the plane $(N_M/(N_B+N_M), (N_M+N_B)/(N_M+N_B+N_{Eu}))$. The gray stripe and axes definitions are the same of Fig. E of this S1 file.

	Year	Europeans	Free Colored	Blacks	Creole	European
Alabama	1820	85451	571	41879	49%	51%
	1830	190406	1572	117549	50%	50%
	1840	335185	2039	253532	50%	50%
	1850	426514	2265	342844	50%	50%
Arkansas	1820	12579	59	1617	28%	72%
	1830	25671	141	4578	33%	67%
	1840	77174	465	19935	33%	67%
	1850	162189	608	47100	33%	67%
Delaware	1810	55361	13136	4177	0%	100%
	1830	57691	15855	3222	0%	100%
Kentucky	1790	61133	114	11839	33%	67%
	1800	179871	741	40343	33%	67%
	1810	324237	1713	80561	33%	67%
	1820	434644	2759	126732	33%	67%
	1830	517787	4917	165213	33%	67%
	1840	590253	7317	182258	33%	67%
	1850	761413	10011	210981	33%	67%
Maryland	1790	208649	8043	103036	33%	67%
	1800	216326	19587	105635	0%	100%
	1810	235117	33927	111502	0%	100%
	1820	260223	39730	107397	0%	100%
	1830	291108	52938	102994	0%	100%
	1840	318204	62078	89737	0%	100%
	1850	417943	74723	96308	0%	100%
Mississippi	1800	5179	182	3489	46%	54%
	1810	23024	240	17088	50%	50%
	1820	42176	458	32814	50%	50%
	1830	70443	519	65659	64%	36%
	1840	179074	1366	195211	67%	33%
	1850	295718	930	309878	67%	33%
Missouri	1810	17227	607	3011	0%	100%
	1820	55988	317	10222	33%	67%
	1830	114795	569	25091	33%	67%
	1840	323888	1574	58240	33%	67%
	1850	592004	2618	87422	33%	67%

Table C: Same as Table A of this S1 file but for those States that did not develop the creole language. Continued on Table D of this S1 file.

	Year	Europeans	Free Colored	Blacks	Creole	European
North Carolina	1790	288204	4975	100572	33%	67%
	1800	337764	7043	133295	33%	67%
	1810	376410	10266	168824	33%	67%
	1820	419200	14612	205017	33%	67%
	1830	472843	19543	245601	33%	67%
	1840	484870	22732	245817	33%	67%
	1850	553028	27463	288548	33%	67%
Pennsylvania	1790	424099	6537	3737	0%	100%
	1830	1309900	37930	403	0%	100%
Tennessee	1790	32013	361	3417	14%	86%
	1800	91709	309	13581	33%	67%
	1810	215875	1317	44535	33%	67%
	1820	339927	2727	80117	33%	67%
	1830	535746	4555	141603	33%	67%
	1840	640627	5524	183059	33%	67%
	1850	756836	6422	239459	33%	67%
Virginia	1790	442115	12766	293427	49%	51%
	1800	514280	20124	345796	46%	54%
	1810	551534	30570	392518	46%	54%
	1820	603087	36889	425153	46%	54%
	1830	694300	47318	469757	33%	67%
	1840	740858	49852	419087	31%	69%
	1850	894800	54333	472528	31%	69%
New Jersey	1790	169954	2762	11423	0%	100%
	1800	195125	4402	12422	0%	100%
	1810	226861	7843	10851	0%	100%
	1820	257409	12460	7557	0%	100%
	1830	300266	18303	2254	0%	100%
	1840	351588	21044	674	0%	100%
	1850	465569	23810	236	0%	100%

Table D: Continues Table C of this S1 file.

County of Virginia	Europeans	Free Colored	Blacks
Augusta	9260	59	1567
Albemarle	6835	171	5579
Accomack	8976	721	4262
Amherst	8286	121	5296
Amelia & Nottoway	6684	106	11307
Botetourt & Montgomery	9241	24	1259
Buckingham	5496	115	4168
Berkley	16650	131	2932
Brunswick	5919	132	6776
Bedford	7725	52	2754
Cumberland	3577	142	4434
Chesterfield	6358	369	7487
Charlotte	5199	63	4816
Culpeper	13809	70	8226
Charles City	2084	363	3141
Caroline	6994	203	10292
Campbell	4946	251	2488
Dinwiddie	6039	561	7334
Essex	3543	139	5440
Elizabeth City	1556	18	1876
Fauquier	11157	93	6642
Fairfax	7611	135	4574
Franklin	5735	34	1073
Fluvanna	2430	25	1466
Frederick Division	15315	116	4250
Gloucester	6225	210	7063
Goochland	4140	257	4656
Greeneville	2530	212	3620
Greenbrier & Kanawa	5676	20	319
Henrico	5600	581	5819
Hanover	6291	240	8223
Hampshire	6879	13	454
Harrison	2013	0	67
Hardy	6556	411	369
Halifax	8931	226	5565
Henry	6763	165	1551
Isle of Wight	4786	375	3867
James City	1519	146	2495
King William	2893	84	5151

Table E: Census data regarding the populations of Europeans (Whites), Free Colored (Mulattos) and Blacks (Bozals) in Virginia's counties and parishes in year 1790. Continued on Table F of this S1 file.

County of Virginia	Europeans	Free Colored	Blacks
King and Queen	4159	75	5143
King George	3123	86	4157
Lunenburg	4547	80	4332
Loudon	14749	183	4030
Lancaster	2259	143	3236
Louisa	3880	14	4573
Mecklenburg	7555	416	6762
Middlesex	1531	51	2558
Monongalia	4602	12	154
Montgomery & Botetourt	12394	6	828
Norfolk	8928	251	5345
Northampton	3181	464	3244
New Kent	2391	148	3700
Northumberland	4506	197	4460
Nansemond	4713	480	3817
Orange	5436	64	4421
Ohio	4907	24	281
Prince Edward	4082	32	3986
Prince William	6744	167	4704
Prince George	3387	267	4519
Powhatan	2286	211	4325
Pendleton	2378	1	73
Pittsylvania	8538	62	2979
Princess Anne	4527	64	3202
Richmond	2918	83	3984
Randolph	932	0	19
Rockingham	6677	0	772
Russell	3143	5	190
Rockbridge	5805	41	682
Spotsylvania	5171	148	5933
Stafford	5465	87	4036
Southampton	6312	559	5993
Surry	2762	368	3097
Shenandoah	9979	19	512
Sussex	4771	391	5387
Warwick	667	33	990
Washington	5167	8	450
Westmoreland	3183	114	4425
York	2115	358	2760

Table F: Continues Table E of this S1 file.

County of Georgia	Europeans	Free Colored	Blacks
Camden	221	14	70
Glyn	193	5	215
Liberty	1303	27	4025
Chatham	2426	112	8201
Effingham	1674	0	750
Richmond	7162	39	4116
Burke	7064	11	2392
Washington	3856	2	694
Wilks	24152	180	7268
Franklin	885	0	156
Greene	4020	8	1377

Table G: Census data regarding the populations of Europeans (Whites), Free Colored (Mulattos) and Blacks (Bozals) in Georgia's counties and parishes in year 1790.

County of South Carolina	Europeans	Free Colored	Blacks
Beaufort	4364	153	14236
Chester	5881	47	938
Claremont	2438	0	2110
Clarendon	1790	0	602
Fairfield	6138	0	1485
Lancaster	4864	68	1370
Richland	2479	14	1437
York	5652	29	923
Berkeley	692	60	5170
Colleton	585	22	4705
Dorchester	1252	25	3022
Christ	445	272	11
St. Andrews	370	31	2546
St. Bartholomes	2133	135	10338
St. James Goose Creek	439	15	2333
St. James Santee	437	15	3345
St. Pauls	216	15	3202
St. Phillips & St. Michaels	8089	586	7684
St. Stephens	226	1	2506
St. Thomas	397	34	3405
Cheraw	7418	59	3229
All Saints	429	1	1795
Prince Fredericks	3418	32	4685
Prince George	5031	80	6651
Abbeville	7505	27	1665
Edgefield	9605	65	3619
Greenville	5888	9	606
Laurens	8210	7	1120
Newberry	8186	12	1144
Pendleton	8731	3	834
Spartanburg	7907	27	866
Union	6430	48	1215
N. Orangenburg	6731	21	4529
S. Orangenburg	5681	149	1402

Table H: Census data regarding the populations of Europeans (Whites), Free Colored (Mulattos) and Blacks (Bozals) in South Carolina's counties and parishes in year 1790.
